# A case of vitiligo secondary to discoid lupus erythematosus treated with tofacitinib

**DOI:** 10.1097/MD.0000000000043118

**Published:** 2025-06-27

**Authors:** Shiyu Jin, Shiwen Wang, Sha Jin, Chenyu Tang, Ping Wang

**Affiliations:** a Department of Dermatology, Hangzhou Third People’s Hospital, Zhejiang Chinese Medical University, Hangzhou, People’s Republic of China; b Department of Dermatology, Hangzhou Third People’s Hospital, Hangzhou Third Hospital Affiliated to Zhejiang Chinese Medical University, Hangzhou, People’s Republic of China.

**Keywords:** discoid lupus erythematosus, JAK inhibitors, tofacitinib, vitiligo

## Abstract

**Rationale:**

Vitiligo is a common autoimmune disorder characterized by melanocyte destruction, leading to depigmented patches. It is often associated with other autoimmune diseases, including thyroid disease and systemic lupus erythematosus. Discoid lupus erythematosus (DLE) is a prevalent form of cutaneous lupus, and both conditions involve the Janus kinase-signal transducer and activator of transcription (JAK-STAT) signaling pathway. However, the co-occurrence of vitiligo secondary to DLE is rare, and therapeutic approaches remain challenging. This case highlights the potential role of tofacitinib, a JAK inhibitor, in treating this dual pathology.

**Patient concerns:**

A 49-year-old male presented with erythematous lesions on his hands, ears, and forearms, without systemic symptoms. Histopathology confirmed DLE. Despite treatment with hydroxychloroquine and tacrolimus, new white spots developed within the DLE lesions, indicating progressive vitiligo secondary to DLE.

**Diagnoses:**

Histopathology and clinical findings, along with skin computed tomography, revealed both DLE and progressive vitiligo. The diagnosis was confirmed based on the presence of depigmented lesions within existing DLE areas.

**Interventions:**

Tofacitinib (5 mg twice daily) was initiated alongside hydroxychloroquine. The patient was monitored over a 12-month period.

**Outcomes:**

After 1 year of continued tofacitinib treatment, the lesions continued to improve without adverse effects, demonstrating the drug’s effectiveness in this complex case.

**Lessons:**

This case illustrates the successful use of tofacitinib in treating vitiligo secondary to DLE, emphasizing the therapeutic potential of JAK inhibition in overlapping autoimmune skin conditions. Further studies are warranted to validate its long-term safety and efficacy in such complex cases. This is a single case report with a short follow-up duration. Lack of immunologic profiling limits broader generalizability. Controlled studies are needed to confirm these findings.

## 1. Introduction

Vitiligo, a multifaceted autoimmune disorder, is a common depigmenting condition, characterized by melanocyte destruction resulting in white patches. This condition profoundly impacts patients’ mental health and quality of life, with a prevalence ranging from 0.5% to 2%. Research has demonstrated that interferon-γ (IFN-γ), secreted by CD8+ T cells, is a critical cytokine that activates the Janus kinase-signal transducer and activator of transcription (JAK-STAT) pathway, playing a central role in vitiligo pathogenesis.^[1]^ Traditional approaches for vitiligo include topical corticosteroids, phototherapy, and calcineurin inhibitors. Discoid lupus erythematosus (DLE) represents the most prevalent form of chronic cutaneous lupus erythematosus, typically manifesting as discoid erythematous plaques with well-defined borders marked by pigmentation and scaling. The central area of these lesions frequently displays atrophy and hypopigmentation.^[2]^ In DLE pathogenesis, type I and type II IFNs are pivotal, primarily through their activation of the JAK-STAT signaling pathway.^[3]^ Conventional treatments for DLE involve topical agents, antimalarials, glucocorticoids, and immunosuppressants.^[2]^

While conventional treatment regimens for vitiligo and DLE are effective for many patients, they may not always be sufficient in cases where the disease is particularly refractory or when side effects become problematic. In such scenarios, alternative treatments such as tofacitinib, a Janus kinase (JAK) inhibitor targeting the JAK-STAT signaling pathway, may present a viable therapeutic option. This proposition is supported by its mechanism of action, which aligns with the pathogenesis of both conditions. Reports on the use of tofacitinib for treating vitiligo and DLE are sparse, with even fewer documenting simultaneous treatment of both. Herein, we present a case of vitiligo secondary to DLE, where the patient’s lesions gradually subsided following treatment with tofacitinib, without any adverse reactions.

## 2. Case presentation

**Figure 1.**
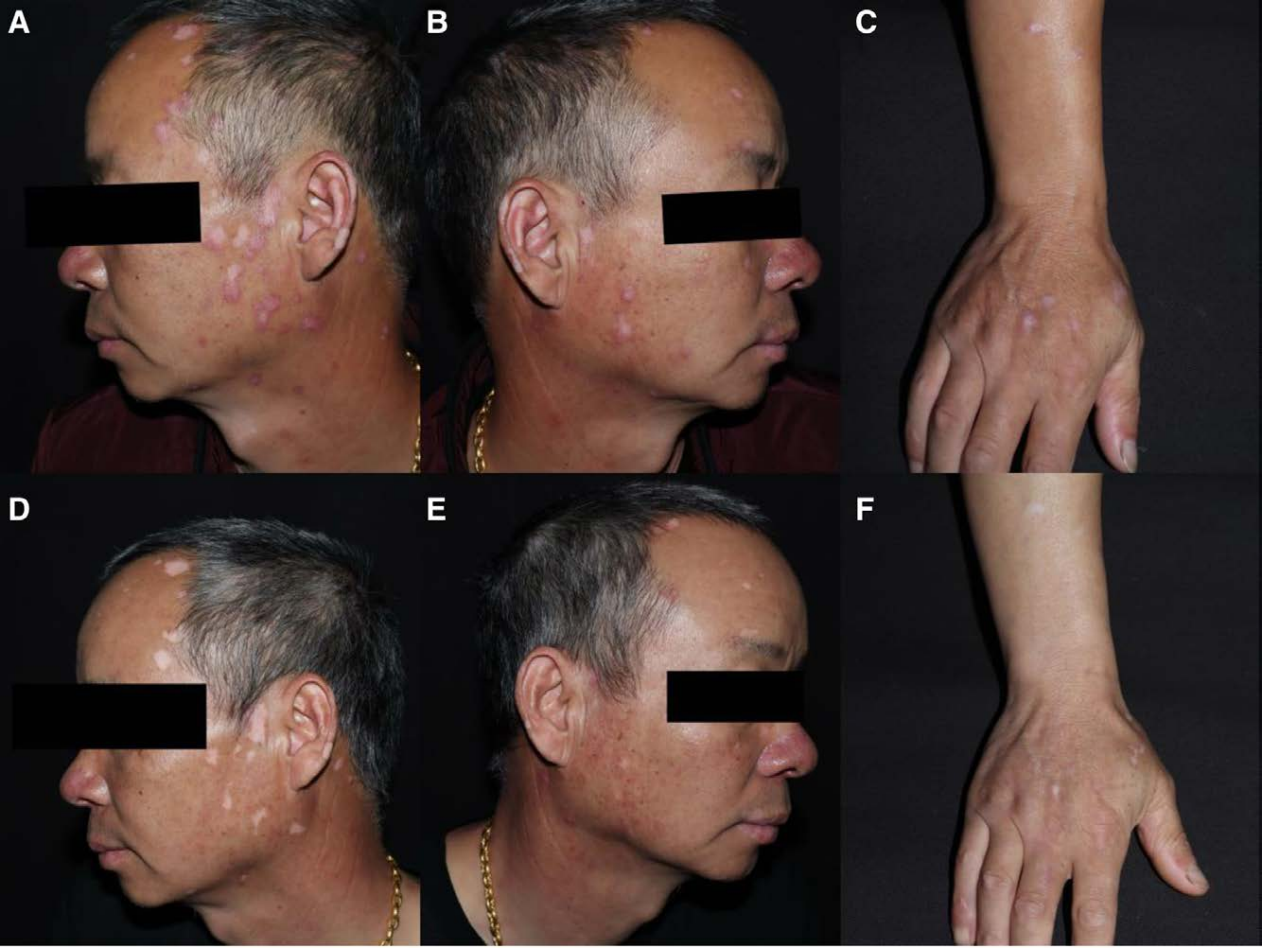
Head and face hand back forearm scattered erythema secondary to white spots for more than 2 months (A–C). After a period of time scattered white spot lesions appeared in the original DLE lesions (D–F).

**Figure 2.**
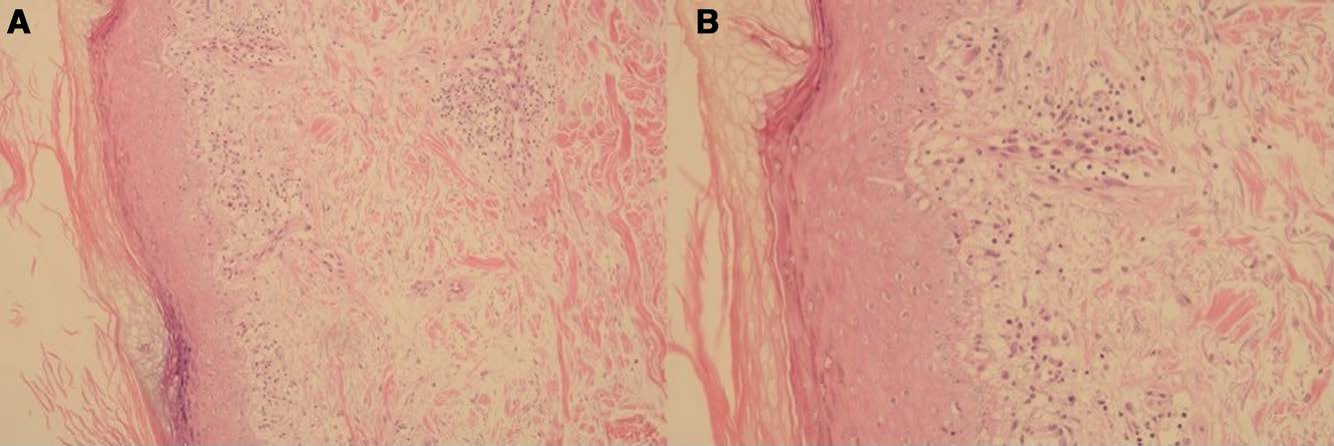
Dermatohistopathology of a lesional skin biopsy (A, B) Hyperkeratosis, focal liquefaction degeneration of basal cells, thickening of basement membrane band, infiltration of a small amount of lymphocytes, histiocytes and plasma cells around blood vessels in the superficial and middle dermis, and loose collagen fibers in the dermis (A: HE × 200, B: HE × 400).

**Figure 3.**
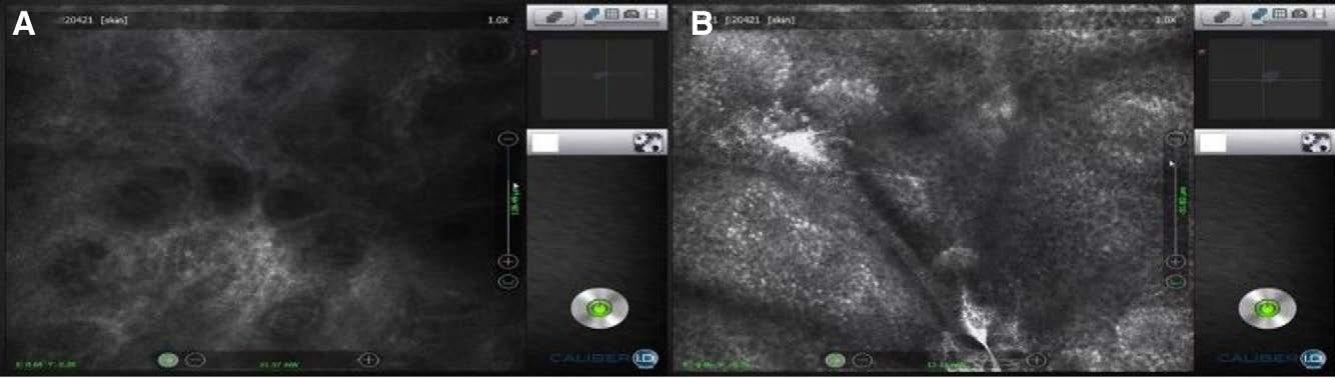
Skin CT (A, B) showed that compared with the surrounding normal skin, the white spine area was atrophied, the pigment ring in the basal layer was missing, and inflammatory cell infiltration was seen in the dermal papilla. CT = computed tomography.

A 49-year-old man presented with a 2-month history of multiple scattered erythema on the dorsal aspect of his hands, ears, forearms without systemic symptoms (Fig. 1A–C). Histopathological examination of the lesion on the left forearm of the patient showed hyperkeratosis, focal liquefaction degeneration of basal cells, dermal mucin deposition, dermal perivascular lymphocyte tissue cell, and plasma cell infiltration (Fig. 2A, B), confirming the diagnosis of DLE. Initially treated with oral hydroxychloroquine 200 mg twice daily and topical tacrolimus, the patient showed no significant improvement. Over time, scattered white spot lesions appeared in the original DLE lesions. Skin computed tomography examination of depigmented spots showed atrophy of the spinous layer in the white spot area, loss of pigment ring in the basal layer, localized residual pigment, and infiltration of inflammatory cells in the dermal papilla (Fig. 3A, B). These findings, combined with clinical assessment, support the diagnosis of progressive vitiligo. Thus, we changed the treatment regimen to tofacitinib 5 mg twice a day plus hydroxychloroquine for 2 months. By the end of the first month of tofacitinib treatment, the erythematous lesions had significantly flattened, with reduced scaling and inflammation. The depigmented areas showed signs of perifollicular repigmentation, and no new lesions were observed (Fig. 1D–F). Over the following year, the vitiligo patches gradually repigmented, with more than 75% improvement in the color match compared with the surrounding skin. The DLE plaques also continued to regress without recurrence. No adverse effects, including hematological or hepatic abnormalities, have been noted during follow-up to date.

## 3. Discussion and conclusions

Vitiligo is well-documented for its broad autoimmune foundation, with its coexistence alongside other autoimmune diseases—such as thyroid disease, inflammatory bowel disease, alopecia areata, systemic lupus erythematosus, and discoid lupus increasingly reported in the literature. Vitiligo secondary to DLE is particularly rare, rendering this case noteworthy. We believe that in this case, the onset of vitiligo-like lesions following DLE suggests a possible pathogenic link between the 2 conditions. Both diseases are known to involve immune dysregulation mediated through the JAK-STAT signaling pathway. While this temporal and mechanistic association supports our hypothesis, we acknowledge that it remains speculative and requires further investigation through larger case series and mechanistic studies. The concurrent manifestation of these diseases implies a complex interplay of autoimmune mechanisms, warranting further exploration of the JAK-STAT pathway’s role in their pathogenesis. There is no specific drug available for treating DLE and vitiligo. Conventional treatments, including corticosteroids, antimalarials, and immunosuppressants, have shown limited efficacy in this case.^[4]^ The successful use of tofacitinib underscores its potential as a therapeutic option in similarly complex presentations, where conventional therapies may fall short.

Although the precise triggers and underlying nature of vitiligo and DLE remain elusive, mounting evidence highlights the JAK-STAT signaling pathway as central to their pathogenesis. DLE is predominantly mediated by type I and II IFNs, especially IFN-α and IFN-β, produced by plasmacytoid dendritic cells, which drive the inflammatory cascade. These IFNs activate the JAK-STAT pathway, inducing gene transcription in target cells.^[3]^ Likewise, vitiligo is intricately tied to the JAK/STAT1 pathway, with IFN-γ, secreted by CD8+ T cells, initiating melanocyte destruction. IFN-γ stimulates keratinocytes to express CXCR3 and its ligands (CXCL9, CXCL10, and CXCL11), which recruit CXCR3+ CD8+ T cells, perpetuating a feedback loop that accelerates melanocyte loss.^[1,5]^

The JAK/STAT pathway plays a crucial role in the pathogenesis of both vitiligo and DLE, making it an attractive therapeutic target. Tofacitinib, an oral small-molecule JAK inhibitor, selectively inhibits JAK1 and JAK3^[6]^ and has shown significant therapeutic efficacy in various immune-mediated diseases, including rheumatoid arthritis, systemic lupus erythematosus, and vitiligo.^[7–9]^ In cases of vitiligo secondary to DLE, tofacitinib has been observed to markedly improve the patient’s condition, further underscoring its potential in treating autoimmune disorders. However, additional clinical trials and long-term follow-up are required to fully establish its safety and efficacy.

This study is limited by its single-case design, which limits generalizability. The lack of posttreatment immunological profiling also limits mechanistic insights. In addition, although clinical improvement was observed, longer follow-up and comparative studies are needed to assess the durability and broader applicability of tofacitinib in such cases.

## Author contributions

**Writing—original draft:** Shiyu Jin.

**Data curation:** Shiwen Wang, Ping Wang.

**Visualization:** Sha Jin.

**Investigation:** Chenyu Tang.
